# Supplementary material for: Machine Learning Made Easy (MLme): A Comprehensive Toolkit for Machine Learning-Driven Data Analysis
Source: bioRxiv. 2023 Jul 4:2023.07.04.546825. Preprint. [Version 1] doi: 10.1101/2023.07.04.546825 (PMC10349995; doi:10.1101/2023.07.04.546825)
Supplement: Supplement 2 [file media-2.pdf]

Tool	*GUI*	*Data Exploration*	*Interactive Visualization*	*Design Custom Pipeline*	*Preprocessing*
**MLme**	✓	✓	✓	✓	Scaling, Data Resampling, Feature Selection
TPOT				✓*	Feature Selection
PennAI	✓				Feature Selection
AutoSklearn 2.0				✓*	Scaling, Imputation, Feature Selection
HyperoptSklearn				✓*	Scaling

*Table S1: Comparison of features between MLme and other similar machine learning automation tools. * = Coding expertise is required. GUI = Graphical User Interface.*

Dataset	***Data type***	***Number of Samples***	***Number of Features***	***Target Class ratio***
CLL	mRNA	136	5000	Male (n=82): Female (n=54)
Cervical cancer	miRNA	58	714	Normal (n=29): Tumor (n=29)
TCGA-BRCA	miRNA	1207	1404	Normal (n=104): Tumor (n=1104)
TCGA-BRCA	mRNA	1219	5520	Normal (n=113): Tumor (n=1106)
PBMC	scRNA-seq	1500	500	CD8 Naive (n=500 cells) : CD14 Monocytes (n=500 cells) : CD16 Monocytes (n=500 cells)
Glass Identification	Oxide content (i.e., Na, Fe, K, etc)	214	10	Glass 1 (70), Glass 2 (76), Glass 3 (17), Glass 5 (12), Glass 6 (10), Glass 7 (29)

Table S2: Example datasets used in this study. CLL = Chronic Lymphocytic Leukemia. TCGA = The Cancer Genome Atlas. BRCA = Invasive Breast Carcinoma. PBMC = Peripheral Blood Mononuclear Cells.
